# GmSPX8, a nodule-localized regulator confers nodule development and nitrogen fixation under phosphorus starvation in soybean

**DOI:** 10.1186/s12870-022-03556-2

**Published:** 2022-04-01

**Authors:** Xinzhu Xing, Hui Du, Zhanwu Yang, Xihuan Li, Youbin Kong, Wenlong Li, Caiying Zhang

**Affiliations:** State Key Laboratory of North China Crop Improvement and Regulation, College of Agronomy, Hebei Agricultural University, Baoding, 071000 China

**Keywords:** Legume plants, Nodules, Biological nitrogen fixation, Phosphorus, SPX proteins, BiFC, Y2H, Transcriptional activity

## Abstract

**Background:**

Biological nitrogen fixation (BNF) is an important nitrogen source for legume plants, and highly efficient nitrogen fixation requires sufficient phosphorus (P). However, the mechanism of maintaining nitrogen fixation of the legume nodules under low P concentration remains largely unknown.

**Results:**

A nodule-localized SPX protein, GmSPX8, was discovered by transcriptome and functional analysis of its role in N_2_ fixation was characterized in soybean nodules. *GmSPX8* was preferentially expressed in nodules and its expression was gradually increased during nodule development. And also the expression pattern was investigated using reporter gene β-glucuronidase (GUS) driven by the promoter of *GmSPX8. GmSPX8* was greatly induced and the GUS activity was increased by 12.2% under P deficiency. Overexpression of *GmSPX8* in transgenic plants resulted in increased nodule number, nodule fresh weight and nitrogenase activity by 15.0%, 16.0%, 42.5%, subsequently leading to increased N and P content by 17.0% and 19.0%, while suppression of *GmSPX8* showed significantly impaired nodule development and nitrogen fixation efficiency under low P stress. These data indicated that GmSPX8 conferred nodule development and nitrogen fixation under low P condition. By yeast two-hybrid screening, GmPTF1 was identified as a potential interacting protein of GmSPX8, which was further confirmed by BiFC, Y2H and pull down assay. Transcript accumulation of *GmPTF1* and its downstream genes such as *GmEXLB1* and *EXPB2* were increased in *GmSPX8* overexpressed transgenic nodules, and in the presence of GmSPX8, the transcriptional activity of GmPTF1 in yeast cells and tobacco leaves was greatly enhanced.

**Conclusions:**

In summary, these findings contribute novel insights towards the role of GmSPX8 in nodule development and nitrogen fixation partly through interacting with GmPTF1 in soybean under low P condition.

**Supplementary Information:**

The online version contains supplementary material available at 10.1186/s12870-022-03556-2.

## Background

Legume plants, such as *Medicago truncatula* and soybean (*Glycine max* (L.) Merr.), could establish symbiotic associations with rhizobia to form nodules for biological nitrogen fixation (BNF). BNF has the capacity to fix atmospheric nitrogen (N_2_) into ammonia, which is essential to meet the requirement for N nutrient during plant growth and development processes [1–4]. The amount of N source, fixed by BNF, is about 50 million tons per year, contributing nearly half of the N provided by manufactured fertilizer [5, 6]. Simultaneously, excessive application of chemical fertilizer in agricultural production results in the deterioration of environmental quality and soil systems. Therefore, BNF is a very valuable alternative to N fertilizer [7].

BNF is a really complex process. To improve BNF ability and efficiency in legumes, and to realize BNF in non-leguminous by bio-engineering measure, numerous studies have been focused on the genetics of BNF, especially in the recent years [8]. In *Medicago truncatula,* SHR-SCR module determined the fate of cortical cell to enable de novo nodule organogenesis [9]. In soybean, *Nodule Number Locus 1* (*GmNNL1*) interacted with NopP effector from *Bradyrhizobium* USDA110 to inhibit nodulation [10]. Under salinity stress, glycogen synthase kinase 3 (GSK3)-like kinase inhibited legume-rhizobia symbiosis through phosphorylating GmNSP1 [11]. All these significant findings would promote our understanding on the legume-rhizobia interactions for better BNF.

Nodule formation was an energy-consuming process, which required a large amount of phosphorus (P) [12–15]. Phosphorus deficiency directly impaired nodule initiation, development and N_2_ fixation. Sufficient P supply significantly promoted soybean nodulation with 63% and 85% increases in nodule number and nodule size. Moreover, under P starvation condition, P content in nodules was much higher than other organs, e.g. shoot, root, and leaves [15–21]. Thus, maintaining a relative level of P in nodules is important to plant growth and BNF [22]. Recently, several P homeostasis related genes have proven to be involved in soybean nodulation [22]. Overexpression of *GmPAP12* increased nodule number, nodule fresh weight, nitrogenase activity and the resultant higher N content under low P condition, while its RNAi transgenic lines displayed impaired nodule development and nitrogen fixation ability [23]. GmPT5, a high-affinity P transporter, controls P transport from roots to nodules, essential for maintaining Pi homeostasis in nodules [22]. Therefore, mining functional genes involved in nodulation would greatly improve our understanding on soybean nodulation, and further promote BNF in agricultural production [6, 24, 25].

Proteins containing SPX (Syg1, Pho81 and Xpr1) domain are vital components in P signaling pathway and P homeostasis in the cell. The SPX domain is named after the conserved domain in the N-terminal of yeast gpa1 (Syg1), yeast phosphatase (Pho81) and the human xenotropic and polytropic retrovirus receptor 1 (Xpr1) [26]. In plants, SPX-containing-proteins could be divided into four subfamilies according to the presence of additional domains: SPX-EXS, SPX-MFS, SPX-RING and SPX [27–31]. Among them, SPX family proteins refer to proteins only containing the SPX domain, which have important role in P signaling pathway in plants [26]. In *Arabidopsis,* there are four members of SPX proteins, AtSPX1-AtSPX4, among which AtSPX1-AtSPX3 are responsive to P starvation in roots and shoots. AtSPX1 interacts with PHR1 and has a cellular P-dependent inhibitory effect on PHR1 [32, 33]. In rice, six SPX proteins (OsSPX1-OsSPX6) were identified responding to P starvation. OsSPX1, an ortholog of AtSPX1, interacts with the OsPHR2 in the nucleus to inhibit phosphate starvation responses [34]. OsSPX4, a cytoplasmic SPX protein, negatively regulate P Signaling by interacting with OsPHR2 and preventing translocation of OsPHR2 to the nucleus [35]. The interaction of OsSPX4 and OsPHR2 is competitively inhibited by transcription factor bHLH6, which regulates P homeostasis by antagonizing SPX4 [36]. In soybean, nine SPX members (GmSPX1-GmSPX9) are characterized, among which GmSPX1 is a negative regulator and GmSPX3 is a positive one in the P signaling network [27, 31]. Although great efforts have been made on the function of SPX proteins responding to P deficiency in plants, their roles and molecular mechanisms of SPX proteins in soybean nodulation have not been well addressed.

In this study, a nucleus-localized SPX protein, GmSPX8, in soybean nodulation was characterized under low P condition*. GmSPX8* was preferentially expressed in nodules. The overexpression and suppression analysis demonstrated that *GmSPX8* was responsible for nodule development and nitrogen fixation under P deficiency in soybean. Interacting proteins of GmSPX8 was hunted and verified.

## Results

### Plant growth and nodule development were impaired under P-deficient treatment in soybean

**Fig. 1:**
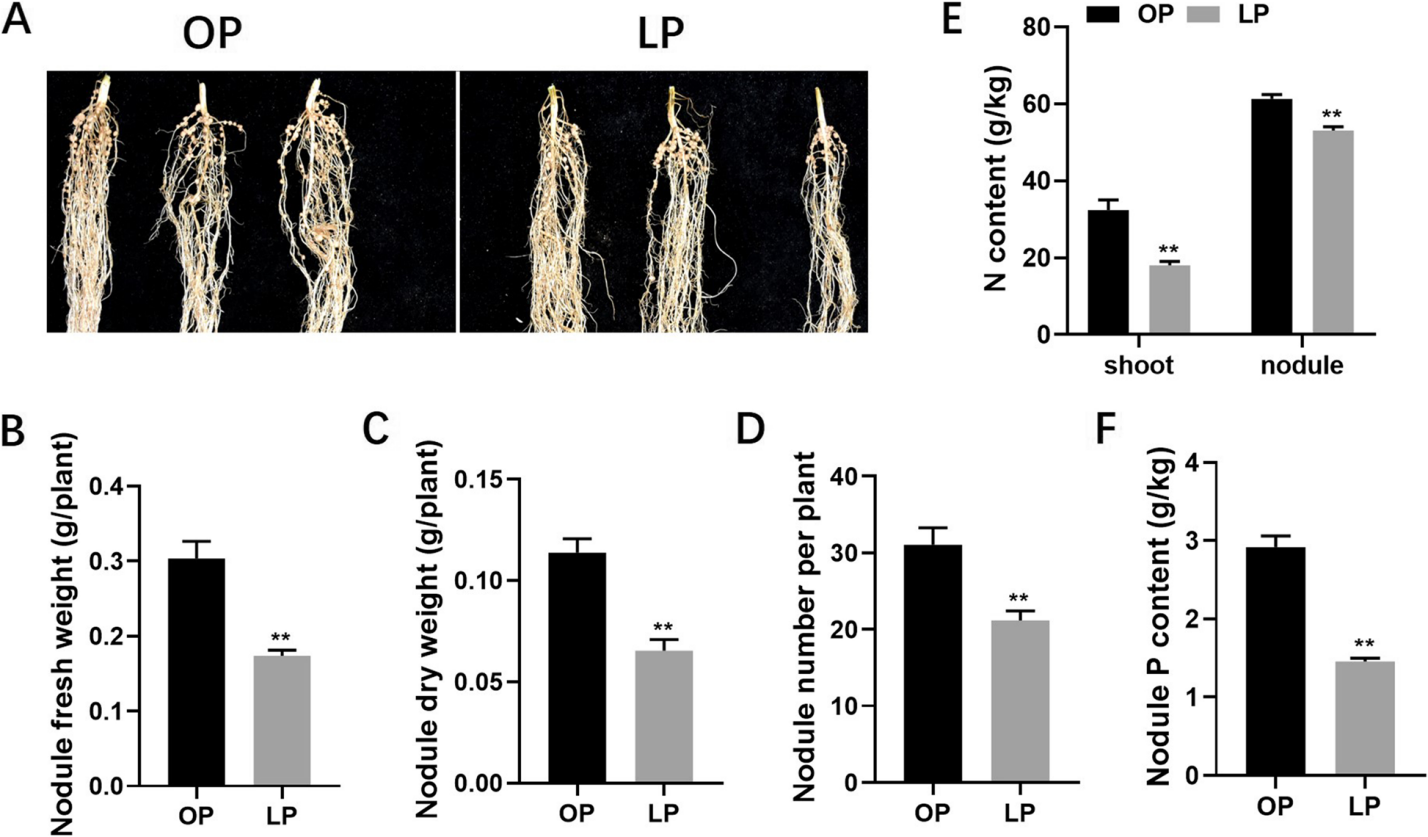
Analysis of soybean nodulation under P-sufficient and P-deficient conditions. **A** Photographs of soybean nodules. **B** Nodule fresh weight. **C** Nodule dry weight. **D** Nodule number. **E** N content of shoot and nodules. **F** P content of nodules. Soybean plants and nodules were harvested at 28 dpi in different P conditions (P-sufficient: OP, 500 μM KH_2_PO4 and P-deficient: LP, 5 μM KH_2_PO4). Data were presented as the average of three different biological replicates and 20 plants for each replicate. Bars showed the means ± SE values. Asterisks indicate significant difference within a P level in t tests. * *p* < 0.05, ** *p* < 0.01

**Fig. 2:**
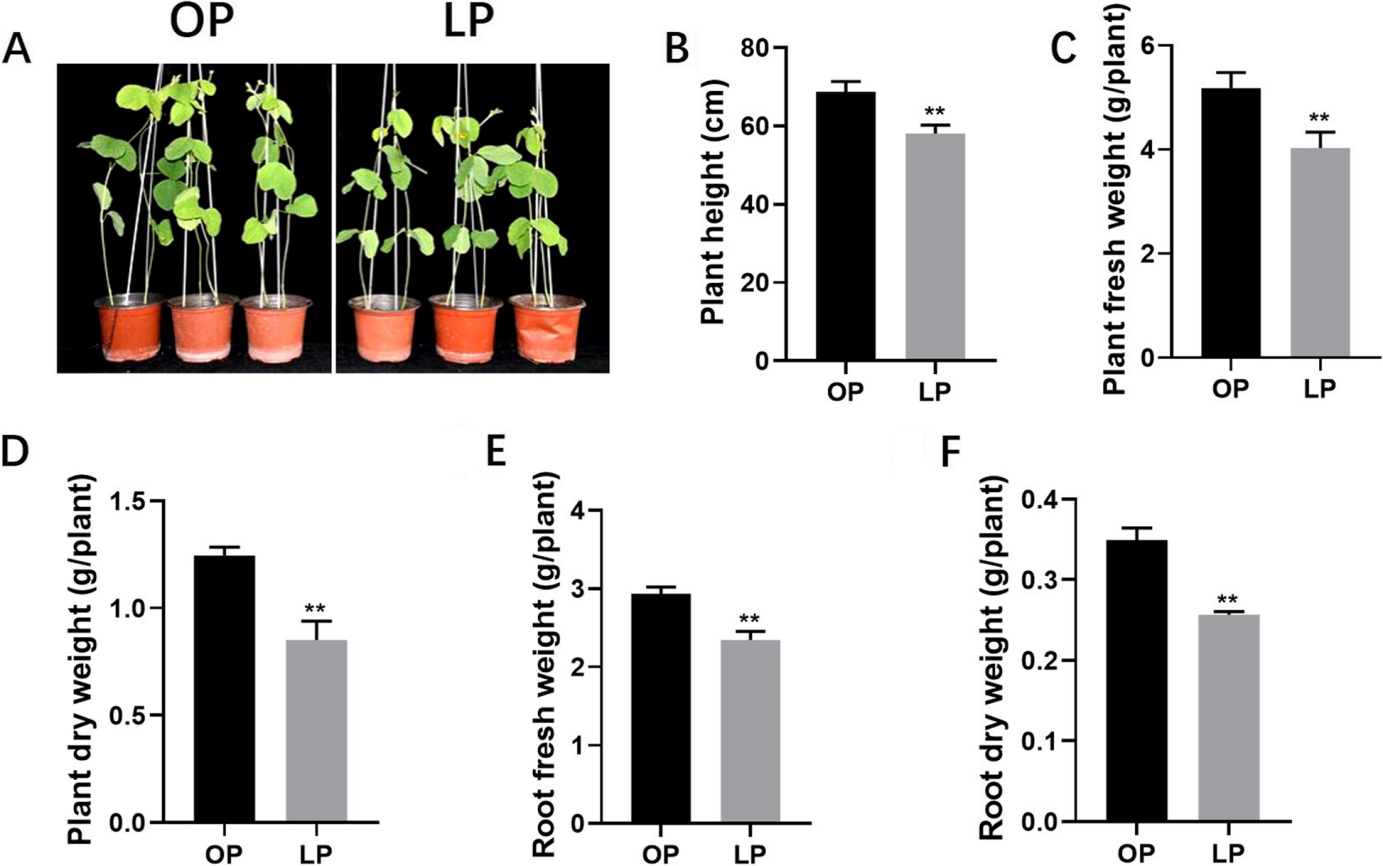
Effects of P deficiency on soybean growth. **A** Phenotype of plant performance. **B** Plant height. **C** Plant fresh weight. **D** Plant dry weight. **E** Root fresh weight. **F** Root dry weight. Soybean seedlings were grown in P sufficient conditions, respectively, with *Bradyrhizobium diazoefficiens* USDA110 inoculation and plants were harvested at 28 dpi. Data are means of four replicates, and error bars show the SE values. Asterisks in B-F indicate significant difference between different P level in t test. * *p* < 0.05, ** *p* < 0.01

Zhonghuang15 (ZH15) was grown under P-sufficient and P-deficient conditions, inoculated with rhizobium *Bradyrhizobium diazoefficiens* USDA110. The phenotype data was collected at 28dpi and analyzed accordingly (Fig. 1). Comparing the data under P-sufficient treatment, the nodule number, nodule fresh-, dry- weight and total P content under low P condition were decreased by 30.1%, 46.6%, 51.6% and 39.8%, respectively. Consequently, the nitrogen fixation was impaired as total N content was reduced by 44.2% and 13.4% in shoot and nodule, respectively (Fig. 1). In addition, plant height, plant fresh- and dry- weight have reductions of 13.0%, 16.0% and 32.0%, respectively, and the root fresh- and dry- weight were simultaneously reduced by 16.9% and 26.5% (Fig. 2). Thus, the nodulation, nitrogen fixation ability and plant growth were significantly impaired in low P condition in soybean.

### Transcriptome analysis of soybean nodules in response to low P stress using RNA-seq

To explore genes responsible for nodule development in response to low P stress, transcriptome dataset of nodules was collected under P-sufficient and P-deficient conditions. Comparing to the treatment of P-sufficient, P deficiency triggered a total of 7723 differentially expressed genes (DEGs) in nodules, with 4382 up-regulated DEGs and 3341 repressed ones (Table S1 and Figure S1A). All the DEGs could be classified into eight biological processes, two cellular components and 20 molecular function terms through Gene ontology (GO) category analysis, and the categories of molecular function contained most DEGs (Figure S1B). These findings suggested that specific regulatory signaling was induced in nodules to maintain nodule formation and development under P deficient condition, and resulted in the differential symbiotic phenotypes of nodules observed under P-sufficient and P-deficient conditions.

### GmSPX8 was preferentially expressed in soybean nodules under low P stress

Given that the SPX domain-containing proteins were reported responsive to the fungus infection in low P stress in soybean [27], eight SPX domain-containing genes among the up-regulated DEGs, *GmSPX1, GmSPX3, GmSPX4, GmSPX7, GmSPX8, GmSPX9, Glyma.03G032400* and *Glyma.10G261900*, were selected for further analysis. Their expression in nodules under P deficient condition was confirmed by qRT-PCR, which was in accordance with their RNA-seq data (Figure S2 and Table S2). Of the eight SPX domain-containing genes, *GmSPX8* presented a highest expression level, implying its role in nodule formation and development.

**Fig. 3:**
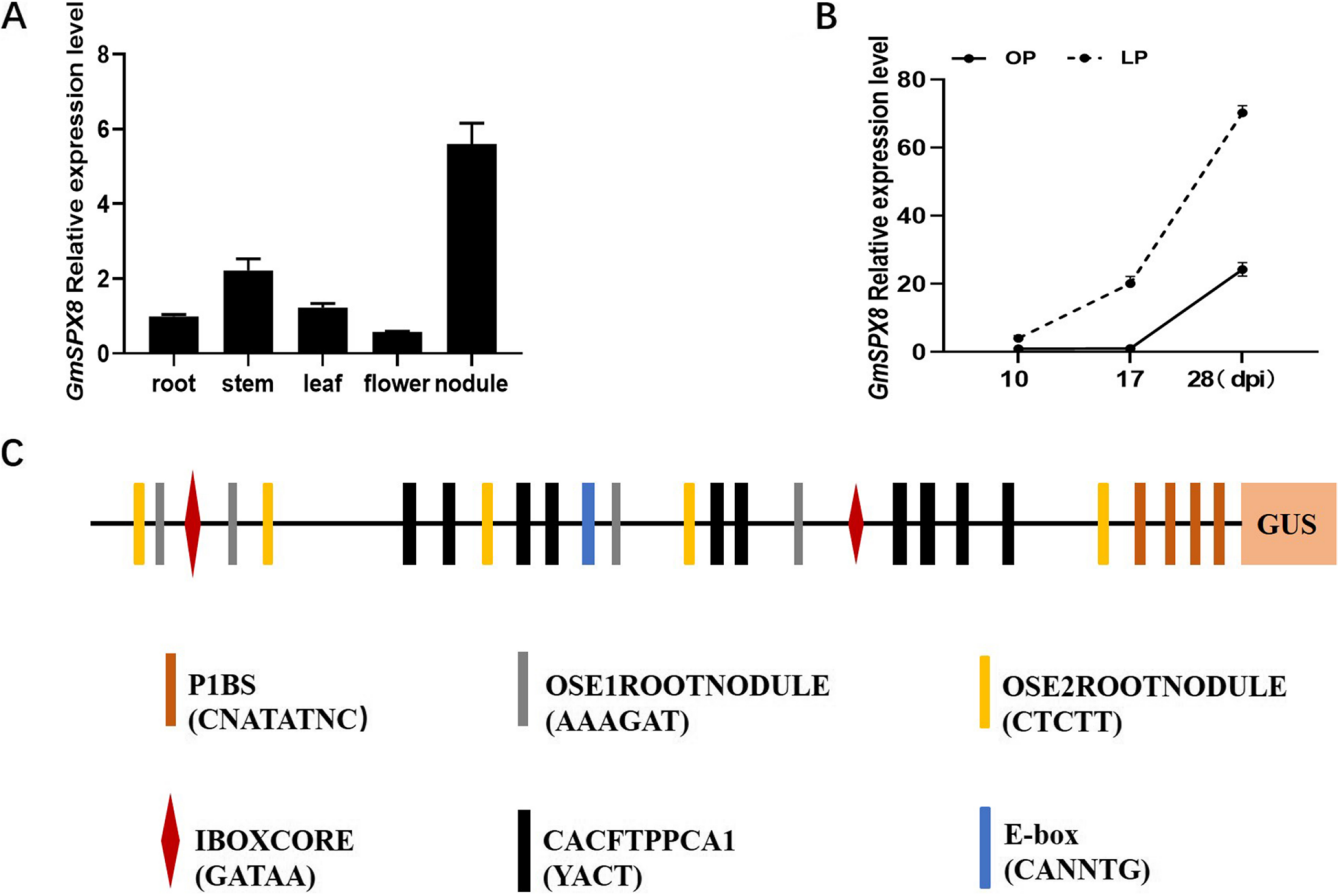
Expression profiles of *GmSPX8* in various tissues. **A** Expression patterns of *GmSPX8* in different organs. **B** Transcript abundance of *GmSPX8* in nodules at different time points determined by qRT-PCR. **C** Schematic representation of the Cis-elements in the promoter of *GmSPX8* analyzed by PlantCARE

The spatial–temporal expression patterns of *GmSPX8* in different soybean organs were assayed via qRT-PCR. This gene mainly expressed in nodules, and its expression was increased gradually during nodule development, while relative low level expressed in root, stem, leaf and flower (Fig. 3A-B).

To understand the expression pattern of *GmSPX8*, its promoter was investigated. Several nodulation and P signaling-related motifs were characterized in its promoter sequence (1500 bp), such as P1BS (PHR1 binding site) element (CNATATNC), E-box (CANNTG), OSE1ROOTNODULE (AAAGAT), and OSE2ROOTNODULE (CTCTT) (Fig. 3C). Visual GUS expression driven by *GmSPX8* promoter was detected in nodules of transgenic composite soybean plants under both P conditions, and the GUS intensity was increased by 12.2% in low P stress than that in P sufficient condition (Figure S3). These data suggested that the *GmSPX8* promoter tends to have a higher activity under P deficient condition.

### GmSPX8 conferred nodule development and nitrogen fixation under low P stress

**Fig. 4:**
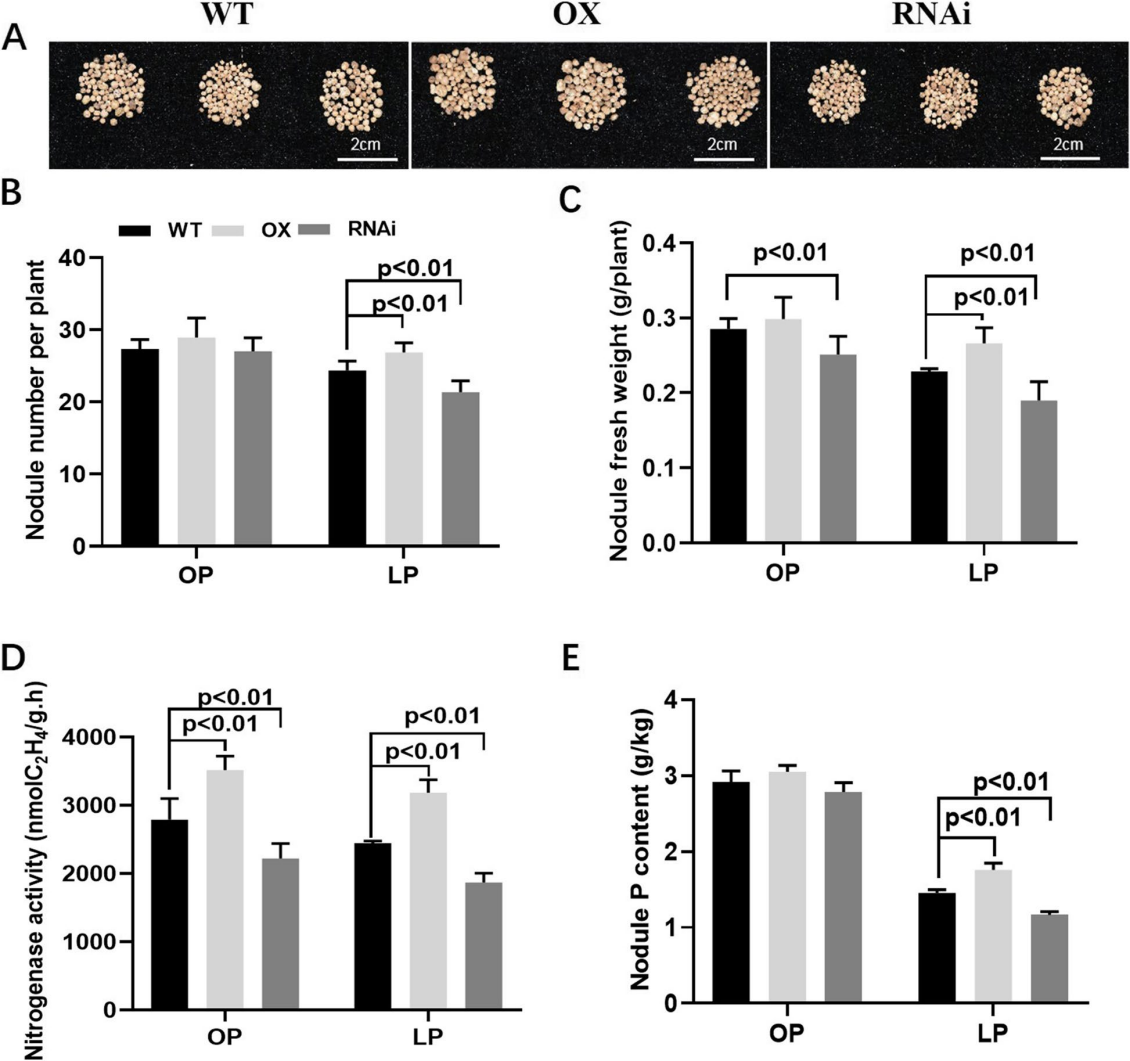
Nodulation analysis of transgenic composite soybean plants either overexpressing (OX) or suppressing (RNAi) of *GmSPX8* under P-sufficient and P-deficient conditions. **A** Growth performance of nodules. **B** Nodule number. **C** Nodule total fresh weight per plant. **D** Nitrogenase activities of nodules measured by acetylene reduction assay. **E** Nodule P content. Transgenic composite soybean plants were harvested at 28 dpi under P-sufficient and P-deficient conditions. Data were presented as the average of three different biological replicates and 20 plants for each replicate. Bars showed the means ± SD values

To further dissect the function of *GmSPX8* in nodule development and nitrogen fixation, transgenic composite soybean plants overexpressing or suppressing (RNAi) of *GmSPX8* were generated and evaluated under P deficient condition (Fig. 4A). Comparing to the non-transgenic wild type (WT), *GmSPX8* overexpressed transgenic plants under low P stress preserved 10.4% and 16.5% more of nodule number and nodule fresh weight, respectively, while *GmSPX8* RNAi plants inversely have fewer nodule number and fresh weight with reductions of 12.3% and 21.9%, respectively. Simultaneously, nitrogenase activity and P contents in *GmSPX8* overexpressed transgenic plants were significantly increased by 37.8%and 35.0% under low P stress, while both were decreased in RNAi plants by 23.6%and 19.8%, respectively (Fig. 4B-E). All these data demonstrated that *GmSPX8* involved in both nodule development and nitrogen fixation under P deficient condition.

### GmSPX8 interacting with GmPTF1 to regulate nodule development and nitrogen fixation under low P stress

**Fig. 5:**
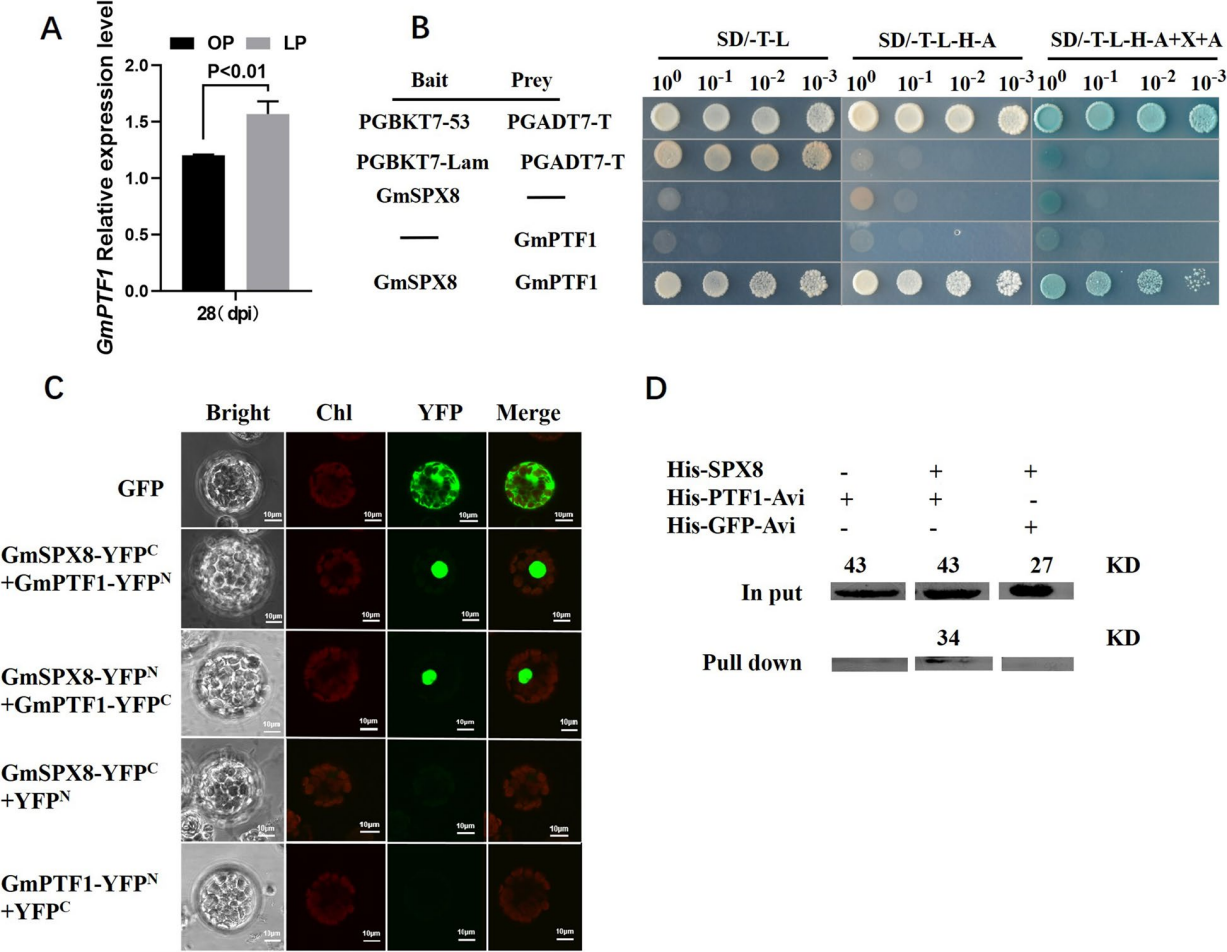
GmSPX8 interacted with GmPTF1 in soybean nodules. **A** Relative expression level of *GmPTF1* in nodules. **B** Interaction between GmSPX8 and Gm PTF1 was shown by Y2H assay. Positive yeast cells containing GmSPX8 and GmPTF1 were selected on SD/-T-L–H-A medium containing 125 ng/ml AbA and 40 ug/ml X-α-Gal. **C** BiFC analysis of the interaction between GmSPX8 and GmPTF1 in *Arabidopsis* protoplasts. GmSPX8-YFP^N^ and GmPTF1-YFP^C^ or GmSPX8-YFP^C^ and GmPTF1-YFP^N^ were cotransformed into *Arabidopsis* protoplasts. The fluorescent signal of YFP was detected by fluorescence microscope. **D** Analysis of interaction between GmSPX8 and GmPTF1 by Pull-down assay using recombinant proteins purified from *E. coli*. All these experiments were repeated three times with similar results

In order to characterize the molecular mechanism of *GmSPX8* involving in soybean nodulation in P-deficient condition, we isolated six potential partners of GmSPX8 from cDNA library of soybean nodules through yeast two-hybrid (Y2H) screening analysis, and GmPTF1 (XM_006588849) was selected for further analysis, due to its nuclear localization and its response to low P stress [37] (Table 1). Under low P condition, *GmPTF1* had a higher abundance in nodules under low P condition than under P-sufficient condition (Fig. 5A). In order to further confirm the physical interaction between GmSPX8 and GmPTF1 in vivo, Y2H assay was performed. Co-transformed with BD-GmSPX8 and AD-GmPTF1, yeast cells grew well on SD/-Trp-Leu-His-Ade + X-α-gal + AbA selective medium, but those co-transformed with BD/AD-GmPTF1 or BD-GmSPX8/AD did not grow on the same medium (Fig. 5B). Bimolecular fluorescence complementation (BiFC) assay in *Arabidopsis* protoplasts also showed that strong yellow fluorescence (YFP) signal was detected in the nucleus when GmSPX8-YFP^N^ and GmPTF1-YFP^C^ or GmSPX8-YFP^C^ and GmPTF1-YFP^N^ were co-expressed in *Arabidopsis* protoplasts (Fig. 5C).The interaction between GmSPX8 and GmPTF1 was also confirmed in vitro through the pull-down assay using recombinant purified proteins in *E.coli* (Fig. 5D). Thus, GmPTF1 interacted with GmSPX8 in soybean nodules under low P stress.

**Table 1:** Interacting partners of GmSPX8 screened by yeast two-hybrid in soybean nodules

ACCESSION	ANNOTATION
XM_003543662	*Glycine max* outer plastidial membrane protein
XM_003526765	*Glycine max* superoxide dismutase
XM_003531923	*Glycine max* transcription factor bHLH93
XM_006588849	*Glycine max* transcription factor bHLH48
XM_003552460	*Glycine max* outer plastidial membrane protein
XM_003554268	*Glycine max* outer mitochondria membrane protein

**Fig.6:**
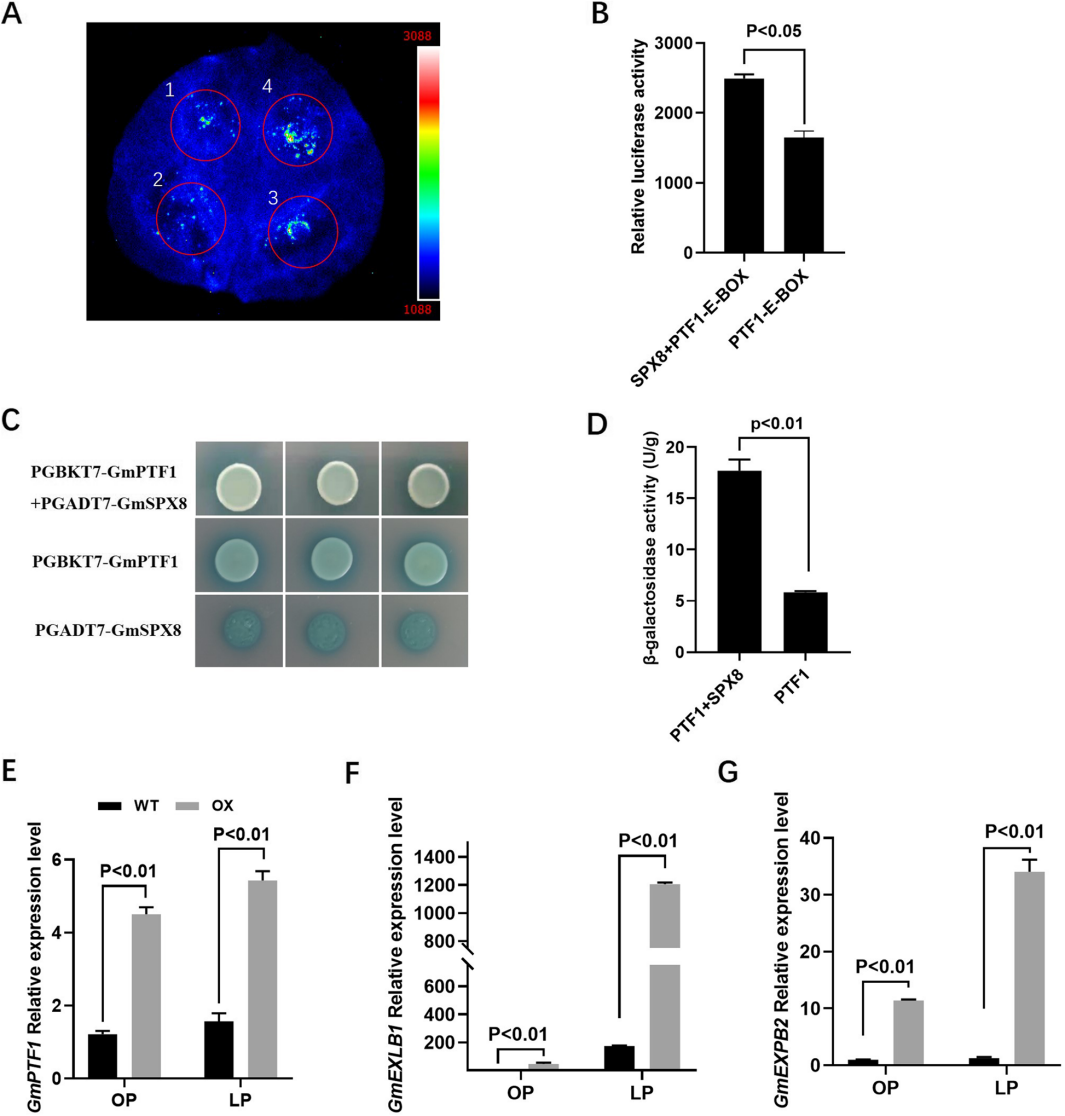
GmSPX8 positively regulated GmPTF1 and its downstream genes. **A** GmSPX8 regulated the transcriptional activity of GmPTF1. 1. PGreenII62-SK empty vector and E-box-LUC, 2. PSK-GmSPX8 and E-box-LUC, 3. PSK-GmPTF1 and E-box-LUC, 4. PSK-GmPTF1 and PSK-GmSPX8 and E-box-LUC. **B** Relative luciferase activity of tobacco leaves expressing the indicated constructs. **C** Effect of GmSPX8 on the transcription activity of GmPTF1 in yeast cells. **D** β-galactosidase activity of yeast cells expressing both GmSPX8 and GmPTF1 or GmPTF1 alone. Expression abundance of *GmPTF1* (**E**), *GmEXBL1* (**F**) and *GmEXPB2* (**G**) in nodules of wild type and *GmSPX8* overexpressed nodules under P-sufficient and P-deficient conditions

To determine GmSPX8 on GmPTF1 transcription activity, transcriptional activation assay was performed using *N. benthamiana* leaves. The transcription activity of GmPTF1 was monitored through the fluorescence intensity of luciferase (LUC). In the presence of GmPTF1, intense LUC signals were observed, and with GmSPX8, the intensity of LUC signals was increased around 50%, which demonstrated that GmSPX8 could enhance the promoter activation activity of GmPTF1 (Fig. 6A-B). In addition, the activation analysis of GmPTF1 was done in yeast cells AH109, containing a β-galactosidase gene as a selection marker (Fig. 6C). In the presence of GmSPX8, the activity of β-galactosidase in yeast cells was two times higher than that of GmPTF1 alone (Fig. 6D). Furthermore, the transcript accumulation of *GmPTF1* was increased more higher in *GmSPX8* overexpressed nodules than in wild type in P-deficient conditions (Fig. 6E). *GmEXPB2* (β-expansin gene), a Cell Wall β-Expansin, was reported to influence soybean nodulation and development, and *GmEXLB1* (expansin-like B1) and *GmEXPB2* were directly regulated by *GmPTF1* [38, 39]. To explore this regulation in nodules, the transcript abundances of *GmEXLB1* and *GmEXPB2* were surveyed in transgenic composite plants with overexpressed *GmSPX8*. *GmEXLB1* and *GmEXPB2* were greatly increased in GmSPX8 overexpressed nodules in P deficient condition (Fig. 6F-G). All these findings suggested that GmSPX8 was involved in soybean nodulation via directly interacting with GmPTF1.

## Discussion

BNF could provide essential N source for legume growth and development, and phosphorus concentration significantly influenced nodule growth and development. Therefore, numerous studies have focused on nitrogen fixation and plant growth under P deficient condition [15–17, 40]. In this study, nodule development was significantly retarded under P deficient condition and subsequently leading to reduced nitrogen fixation ability and N content and P content. Accordingly, plant morphogenesis, e.g. plant height, fresh weight and dry weight, was influenced (Fig. 1–2).

Previous studies have mainly focused on isolating PSI (phosphorus starvation induced) genes and unraveling phosphorus signaling networks in nodules of legume plants through transcriptome and metabolome profiling, e.g. common bean (*Phaseolus vulgaris*), *Medicago truncatula* and soybean. Purple acid phosphatases (PAPs), phosphate transporters (PTs), MYB (v-myb avian myeloblastosis viral oncogene homolog) and bHLH (basic/helix-loop-helix) proteins transcription factors had been reported to involve in Pi homeostasis in nodules [17, 25, 41]. In *Arabidopsis*, soybean and rice, SPX proteins were considered as an important P sensor [31, 33, 34]. GmSPX8 in soybean responded to P starvation in leaves and roots, and was up-regulated by P starvation in mycorrhizal nodules of soybean, implicating its involvement in P homeostasis in soybean nodules [27, 31].In this study, to isolate PSI genes in soybean, transcriptome profiling was conducted with nodules under different P conditions, and eight SPX domain-containing proteins were identified (TableS1 and Figure S2). Of the eight proteins, *GmSPX8* was preferentially expressed in nodules inoculated with *Bradyrhizobium diazoefficiens* USDA110, and its expression level was gradually increased during nodule development (Fig. 3A-B), which was observed for the first time. The promoter analysis revealed that several nodulation- and P deficiency-related motifs presented in *GmSPX8* promoter region, and higher promoter activity was observed in transgenic nodules (Fig. 3C and Figure S3). Furthermore, overexpression of *GmSPX8* in transgenic composite nodules increased nodule number, nodule fresh weight, nitrogenase activity and increased P content in nodules under low P condition, whereas suppression of *GmSPX8* inversely decrease nodule number, nodule fresh weight, nitrogenase activity and decrease P content in nodules (Fig. 4). All these data provided that *GmSPX8* played a vital role in nodulation and P homeostasis of nodules in soybean.

There are several studies on the molecular mechanism of SPX proteins involving in P signaling in plants [34]. OsSPX1 and OsSPX2 interacted with OsPHR2 to inhibit P starvation response in a P-dependent manner in planta [34]. OsbHLH6 interacted with OsSPX4 for Pi signaling and homeostasis in rice [36]. *OsPTF1,* belonging to bHLH family, was induced under low P condition in roots and its overexpression increased the tolerance to Pi starvation in rice [42]. Moreover, *GmPTF1* responded to P starvation primarily through regulating the expression of GmEXPB2 in soybean [43]. In our study, GmPTF1 was isolated as a potential target of GmSPX8 from soybean nodules and overexpression of *GmSPX8* increased the expression of *GmPTF1* and its downstream PSI genes, such as *GmEXPB2*and *GmEXLB1* (Table 1 and Fig. 6E-G). In addition, GmSPX8 could enhance the transcriptional activation activity of GmPTF1 in yeast cells and tobacco leaves (Fig. 6A-D). Taken together, these findings indicate that GmSPX8 responds to P starvation by regulating GmPTF1 and its downstream PSI genes to maintain P content for nodule development and BNF in soybean.

legumes require high energy such as ATP during nodulation and biological N_2_ fixation processes, thus P requirement was high, and also these processes were inhibited through a feedback loop formed by the release of free P in BNF, especially under excessive P conditions [12, 15, 44]. PTF1 could bind to the E-box (CANNTG) element in the promoter regions of regulated downstream genes in *Arabidopsis*, maize and soybean [43, 45, 46]. Here, we found that the E-box element presented in the promoter region of *GmSPX8*, and transcriptional activation assay confirmed the binding of GmPTF1 to the E-box of *GmSPX8* (Fig. 6 A-D). All these data implied that GmPTF1 might regulate the expression of *GmSPX8* to maintain the stabilization of P through a feedback regulation in soybean nodules under P starvation.

## Conclusions

Our data showed that *GmSPX8* is preferentially expressed in soybean nodules under P deficiency. The functional analysis of *GmSPX8* in transgenic composite soybean plants demonstrated that *GmSPX8* conferred to nodule development and nitrogen fixation under low P condition. GmSPX8 interacts with GmPTF1 in nodules and overexpression of *GmSPX8* increased transcription accumulation of *GmPTF1* and its downstream genes. All these findings show that *GmSPX8* regulates nodule development and nitrogen fixation through its interaction with GmPTF1 in soybean under low P condition.

## Materials and methods

### Plant materials and growth conditions

Soybean (*Glycine max (L.) Merr.*) seeds used in this study are originally obtained from State Key Laboratory for North China Crop Improvement and Regulation, Hebei Agricultural University. A genotype of soybean Zhonghuang 15(ZH15) was used in this study for phenotypic and functional analysis. Soybean seeds were surface sterilized and germinated in Petri dishes with wet and sterile filter papers for three days under dark conditions in a growth chamber (28℃, 16/8 h light/dark photoperiod). One-week seedlings were inoculated with rhizobia strain *Bradyrhizobium diazoefficiens USDA110*, and planted into vermiculite watered with nitrogen-free nutrient solution containing 5 μM (P-deficient condition: LP) or 500 μM (P-sufficient condition: OP) of KH_2_PO4. Nodules at 28 days post inoculation (dpi) with rhizobia are mature and have high nitrogen fixation ability [47, 48]. Soybean plants and nodules were separately harvested at 28 dpi for measuring fresh weight, dry weight, height of shoot, total P and N content, nodule number, and nitrogenase activity.

For spatial expression analysis of selected genes responding to P supply and rhizobia inoculation, soybean seedlings were inoculated with *Bradyrhizobium diazoefficiens USDA110*, and then transplanted into vermiculite watered with nitrogen-free nutrient solution, which contained 5 μM (P-deficient condition: LP) or 500 μM (P-sufficient condition: OP) of KH_2_PO4. Shoots, leaves, roots and mature nodules were harvested separately at 28 days after inoculation. Nodules at different developmental stages were separately harvested at 10, 17, 28 day post inoculation. All tissues were frozen in liquid nitrogen and stored at -80℃ for further RNA extraction and qRT-PCR analysis.

### RNA isolation and RNA-seq analysis

Nodule samples were collected from three independent biological replicates for different Pi treatment. Samples were ground in liquid nitrogen and subjected to total RNA extraction using Trizol reagent (Invitrogen, USA). mRNA was purified from total RNA using poly-T oligo-attached magnetic beads (TIANGEN BIOTECH). First strand cDNA was synthesized using random hexamer primer and M-MuLV Reverse Transcriptase (RNase H-) (TIANGEN BIOTECH). Second strand cDNA synthesis was subsequently performed using DNA Polymerase I and RNase H (TIANGEN BIOTECH). These cDNA libraries were sequenced on an Illumina Novaseq6000 platform and 150 bp paired-end reads were generated. Feature Counts v1.5.0-p3 was used to count the read numbers mapped to each gene, and FPKM [49] of each gene was calculated based on the length of the gene and read counts mapped to this gene. Differential expression analysis was performed using the DESeq2 R package (1.16.1), which provides statistical routines for determining differential expression in digital gene expression data using a model based on the negative binomial distribution. The resulting *P*-values were adjusted using the Benjamini and Hochberg’s approach for controlling the false discovery rate. Genes with an adjusted P-value < 0.05 were assigned as differentially expressed [49–51].

### Quantitative real-time PCR (qRT-PCR)

For qRT-PCR, total mRNA was isolated and cDNA was synthesized accordingly. The specific primers for qRT-PCR are shown in Table S3. The qRT-PCR condition was: 30 s at 95℃, followed by 40 cycles of 5 s at 95℃, 15 s at 60℃ and 12 s at 72℃, and a final 5 s at 72℃. qRT-PCR was performed using SYBR Premix EX Tag™ (TaKaRa) on a CFX96™ real-time system (Bio-rad). The cycle threshold (CT) values of each sample were standardized using *GmActin11* and the relative fold change (FC) of gene expression was calculated based on the 2^−ΔΔCT^ method [52].

### Construction of GmSPX8 overexpression and RNAi cassettes and soybean hairy root transformation

For overexpression construct, full-length ORF of *GmSPX8* was cloned into pCAMBIA1390 under *CaMV 35S* promoter between *BamHI* and *PstI* enzyme sites. For *RNAi* constructs, about 200 bp fragment specific to *GmSPX8* was cloned into pTCK-303 vector between BamHI and KpnI, SpeI and SacI, respectively, under *CaMV* 35S promoter [49]. Hairy root transformation through *Agrobacterium rhizogenes* strain K599 and determination of GUS positive roots were performed as previously described [50]. Transgenic composite plants were inoculated with *Bradyrhizobium diazoefficiens USDA 110* and grown in different P conditions. Nodule samples were harvested at 28 day after rhizobium inoculation and analyzed [50, 51].

## Acetylene reduction assay

Nitrogenase activity was measured by Acetylene Reduction Assay with the available protocol [53].

## Measurement of N and P contents

Dried samples were ground and digested with HNO_3_ in a microwave oven. The resulting samples were subjected to themeasurement of N and P content. P content was measured by the color reaction of P-molybdate blue at the obsorbance of 700 nm, and N content was determined using semimicro-kjeldahl determination method in a nitrogen analyzer [23, 54].

## Yeast two-hybrid assay

The ORF of *GmSPX8* as bait was cloned into pGBKT7-BD vector and then transformed into the yeast strain Y2H Gold. Interacting proteins of *GmSPX8* was screened from yeast cDNA library of soybean nodules using Matchmaker Gold Yeast Two-Hybrid System (Clontech, 630,489, USA) following the manufacturer’s instruction. pGBKT7-53 and pGADT7-T were used as positive control, pGBKT7-Lam and pGADT7-T were used as negative control. The Y2H assay was biologically repeated three times.

## Bimolecular fluorescence complementation (BiFC) analysis

Full length CDS of *GmSPX8* and *GmPTF1* were cloned into vector p326YFP^N^ and p326YFP^C^, respectively, to generate GmSPX8-YFP^N^, GmPTF1-YFP^C^, GmSPX8-YFP^C^ and GmPTF1-YFP^N^ [55]. The resulting constructs were then co-transformed into *Arabidopsis* protoplasts by polyethylene glycol (PEG)-mediated transformation as described previously [56]. YFP fluorescence was imaged using a confocal microscope.

## Expression and purification of fusion proteins and in vitro pull-down assays

Full length CDS of *GmPTF1* was cloned into pET-28a ( +) vector containing an His-tag in the amino-terminus, and *GmSPX8* was cloned into a modified pET-28a ( +) vector with an additional Avi-tag at the C-terminal end (*His-GmSPX8-Avi*) [57]. The resulting constructs were introduced into *Escherichia coli* strain BL21 (DE3) (EMD Chemicals, Gibbstown, NJ) with or without birA (encoding biotin protein ligase) for biotinylation [58]. Recombinant proteins were induced by 0.5 mM isopropyl-β-d-thiogalactoside for 4 h at 28 °C, and *GmSPX8* was purified by affinity chromatography using streptavidin agarose resin (Thermo Fisher Scientific, Waltham, MA). The pull-down assays were performed as described previously [35].

## Construction of *pGmSPX8-GUS*cassette, histochemical GUS staining and activity assay

The 1500-bp promoter fragment of *GmSPX8* was cloned into the PcamG vector between *SacI* and *SalI* restriction enzyme sites to make *pGmSPX8-GUS* construct [59]. For GUS staining, transgenic soybean root nodules were incubated at 37 °C for 12 h in 5-bromo-4-chloro-3-indolyl-β-D-glucuronic acid (X-Gluc) solution [60], and then the root tissues were washed with ethanol (70% v/v) before photographing. For GUS activity, total nodule proteins were extracted and incubated in a mixture containing 10 mM 4-methylumbelliferyl β-D-glucuronide (MUG; Sigma, USA) for 1 h at 37 °C. The fluorescence product of 4- methylumbelliferone (4-MU) was monitored using a Versa Fluor Fluorometer (Bio-Rad) with excitation at 365 nm and emission at 455 nm. The assay was repeated at least three times, and the data was calculated as the mean of independent experiments with the respective standard deviation.

## Transcription activation assay in yeast cells

The Matchmaker Gold Yeast Two-Hybrid System (Clontech, Mountain View, CA, USA) was used to test GmPTF1 transcriptional activation activity. *GmPTF1* and *GmSPX8* were cloned into pGBKT7-BD and pGADT7-AD vector, respectively to generate pGBKT7-BD-GmPTF1 and pGADT7-AD-GmSPX8 constructs. pGBKT7-BD-GmPTF1 and pGADT7-AD-GmSPX8 or pGBKT7-BD-GmPTF1 alone were transformed into yeast strain AH109, and the yeast cells were grown in SD/-Trp-His + X-α-gal plates to assay the activation ability of GmPTF1 in yeast.

## Transcriptional activity assays in tobacco

Full length ORF of GmSPX8 and GmPTF1 were cloned into the entry vector PGreenII62-SK (PSK-GmSPX8 and PSK-GmPTF1). A 186 bp length of promoter of GmSPX8 containing E-box (CAAATG) was fused to the LUC reporter gene on the PGreenII0800-LUC vector (E-box-LUC) [61]. The resulting constructs were introduced into *A. tumefaciens* bacteria GV3101 (Psoup-P19) and transiently transformed into the abaxial side of leaves of 4-week-old *N. benthamiana* plants. After 2 d of inoculation, the infiltrated leaves were harvested and sprayed with 1 mM D-luciferin. The fluorescence was detected after 10 min using a plant imaging system (Tanon-5200Multi; Tanon, Shanghai, China). The images were analyzed by imagej software (National institutes of Health, China).

## Statistical methods

Statistical analyses were performed using SPSS 17.0 software (IBM, United States).

## Supplementary Information

**Additional file 1:** **Figure S1. **(**A**) The volcano plot showing DEGs. (**B**) GOanalysis.

**Additional file 2:** **Figure S2.** Heatmap presentation of expression of candidategenes obtained from RNA-seq data under P-sufficient and P-deficient conditions.(**A**) Expression of selected genesfrom RNA-seq data analysis. (**B**) Validationof the expression from RNA-seq data by quantitative real-time PCR (qRT-PCR).OP: P sufficient condition. LP: P deficient condition.

**Additional file 3:** **Figure S3. **(**A**) Expression pattern of GmSPX8 intransgenic nodules harboring P_GmSPX8_-GUS construct. Transgeniccomposite soybean plants were grown in different P conditions and nodules wereharvested at 28 dpi for GUS staining. (**B**)GUS activity of transgenic nodules driven by the promoter of GmSPX8. Values aremeans of 10 independent lines for each P treatment. Bars showed the means ± SDvalues

**Additional file 4:** **Table S1. **Transcriptome data showing all the DEGs.

**Additional file 5:** **Table S2. **Cycle threshold (CT) Value of RT-PCR.

**Additional file 6:** **Table S3. **Primers used in this study.

## Data Availability

All the data used in this study are included in this published article and its additional files. The RNA-seq data can be found in the NCBI SRA database with the accession PRJNA739502. Our SRA record, https://www.ncbi.nlm.nih.gov/sra/PRJNA739502 could be accessible upon this publication in BMC Plant Biology.

## Abbreviations

BNF: Biological Nitrogen Fixation
ORF: Open Reading Frame
cDNA: Complementary DNA
GUS: β-Glucuronidase
N: Nitrogen
P: Phosphorus
qPCR: Real-time quantitative PCR
Trp: Trptophan; Leu: Leucine
His: Histidine
Ade: Adenine
ABA: Aureobasidin A
WT: Wild Type
OX: Overexpression
DPI: Days Post Inoculation
