# Supplementary figures and images for: GmSPX8, a nodule-localized regulator confers nodule development and nitrogen fixation under phosphorus starvation in soybean
Source: BMC Plant Biol. 2022 Apr 1;22:161. doi: 10.1186/s12870-022-03556-2 (PMC8973899; doi:10.1186/s12870-022-03556-2)

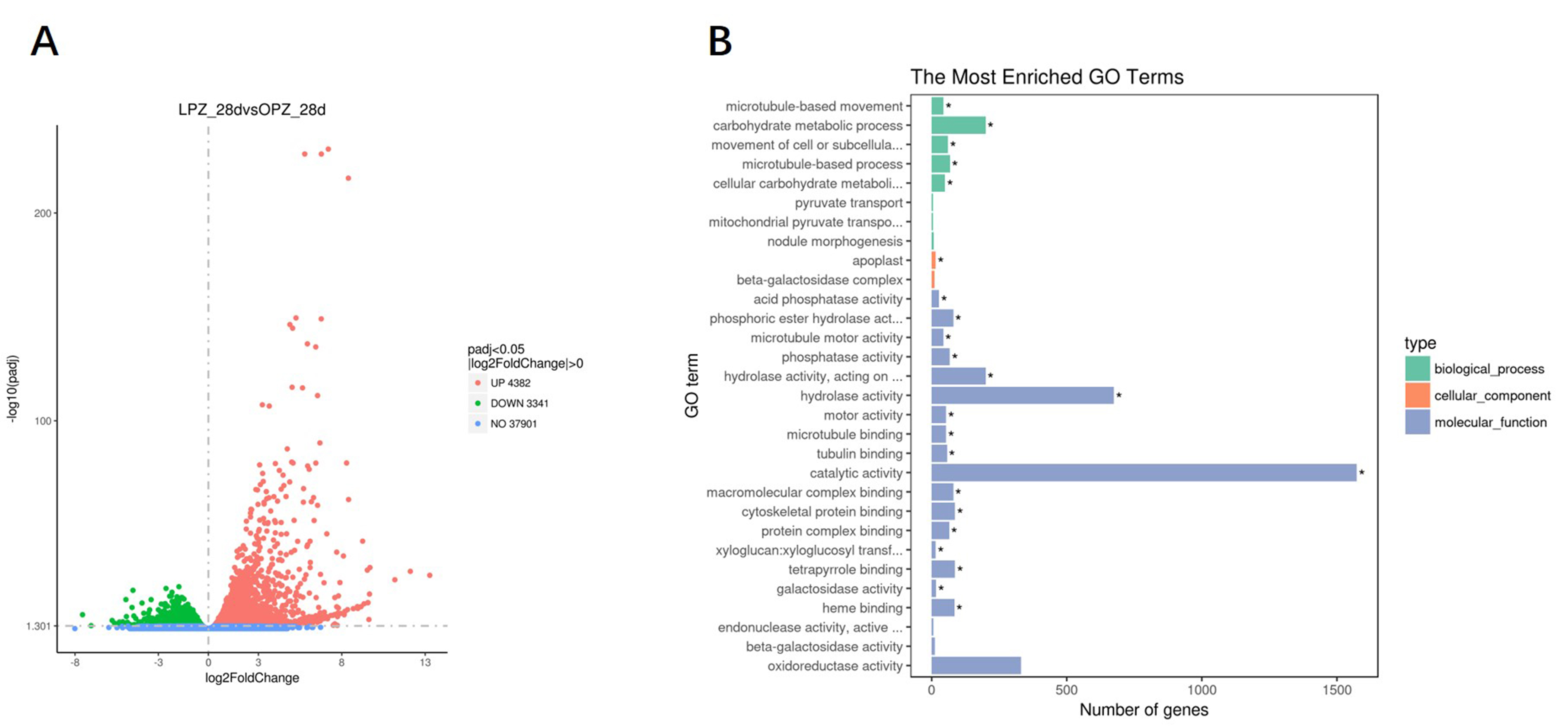

Supplement: Supplementary file 1 — Additional file 1: Figure S1. (A) The volcano plot showing DEGs. (B) GOanalysis. [file 12870_2022_3556_MOESM1_ESM.jpg]

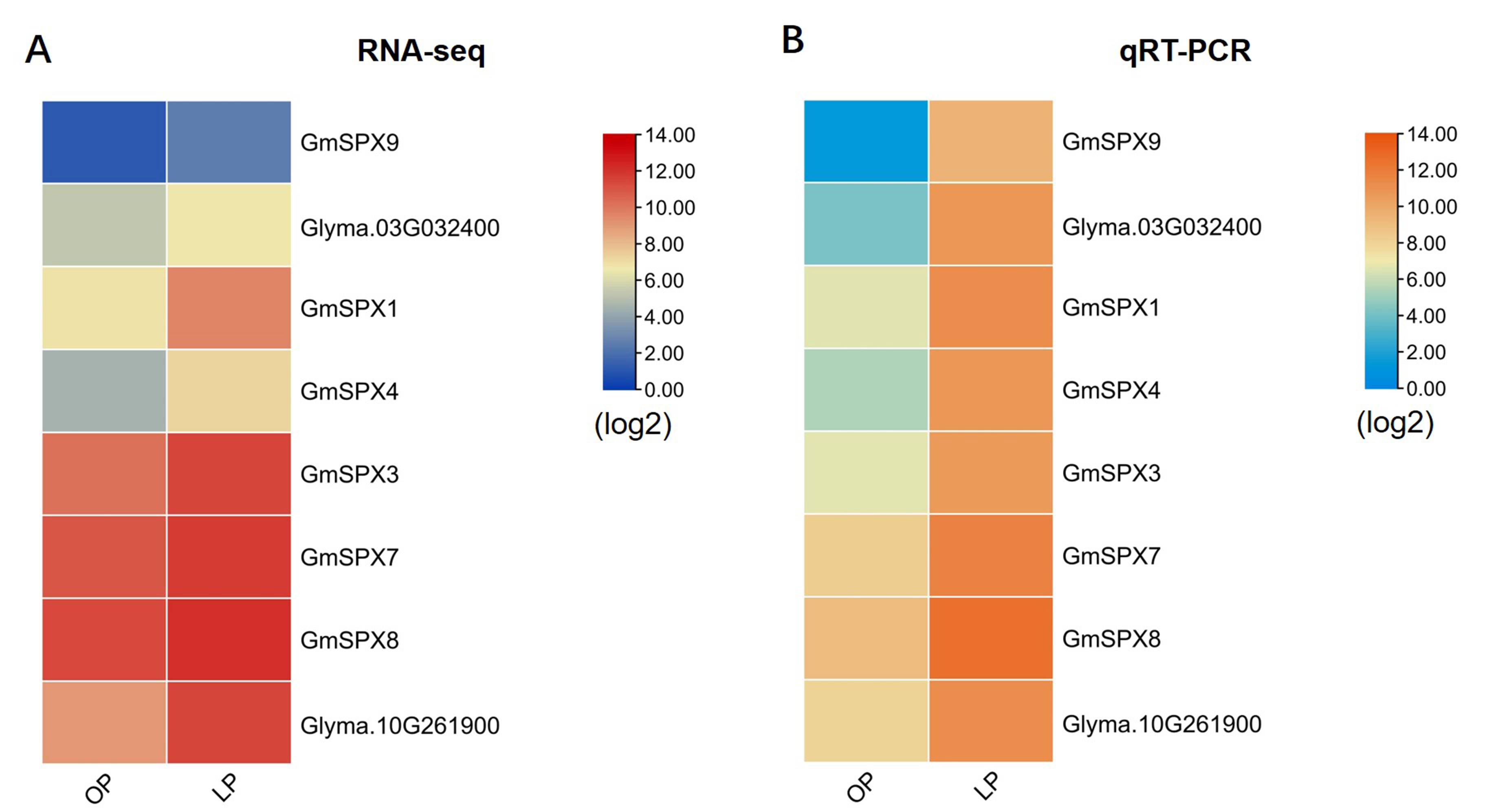

Supplement: Supplementary file 2 — Additional file 2: Figure S2. Heatmap presentation of expression of candidategenes obtained from RNA-seq data under P-sufficient and P-deficient conditions.(A) Expression of selected genesfrom RNA-seq data analysis. (B) Validationof the expression from RNA-seq data by quantitative real-time PCR (qRT-PCR).OP: P sufficient condition. LP: P deficient condition. [file 12870_2022_3556_MOESM2_ESM.jpg]

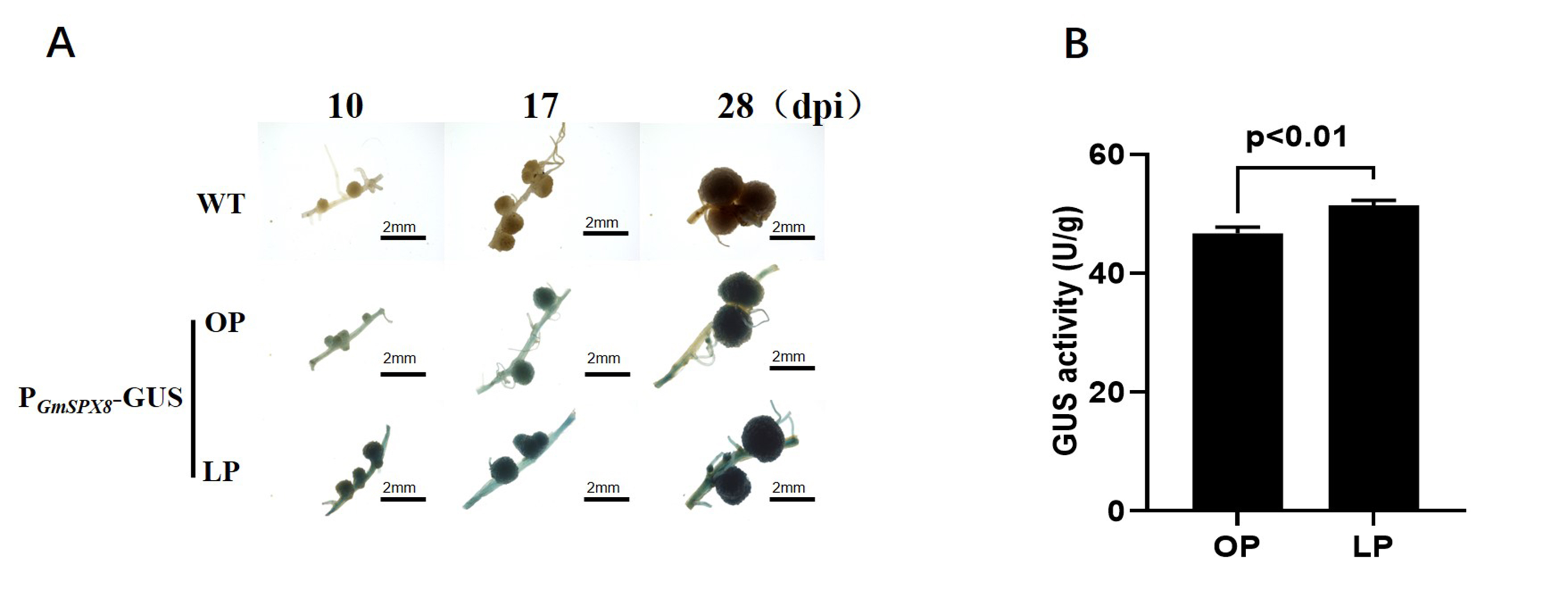

Supplement: Supplementary file 3 — Additional file 3: Figure S3. (A) Expression pattern of GmSPX8 intransgenic nodules harboring PGmSPX8-GUS construct. Transgeniccomposite soybean plants were grown in different P conditions and nodules wereharvested at 28 dpi for GUS staining. (B)GUS activity of transgenic nodules driven by the promoter of GmSPX8. Values aremeans of 10 independent lines for each P treatment. Bars showed the means ± SDvalues [file 12870_2022_3556_MOESM3_ESM.jpg]
