# Supplementary material for: GmSPX8, a nodule-localized regulator confers nodule development and nitrogen fixation under phosphorus starvation in soybean
Source: BMC Plant Biol. 2022 Apr 1;22:161. doi: 10.1186/s12870-022-03556-2 (PMC8973899; doi:10.1186/s12870-022-03556-2)
Supplement: Supplementary file 6 — Additional file 6: Table S3. Primers used in this study. [file 12870_2022_3556_MOESM6_ESM.docx]

Table S1. Primers used in this study

| Function |  |  |
| --- | --- | --- |
| RT-PCR | 01-F | GCCCCAGTTGAATGCCAAAG |
|  | 01-R | CGGCTTCGACAACTCTGTCT |
|  | 02-F | CACAGTTGCGGCTTTGCTTA |
|  | 02-R | TAGCAAGTTGAGTGGTGGCA |
|  | 03-F | AGTGTTGGAGCAGCCCTTTT |
|  | 03-R | CTGCCACAGTTATCGCCTCT |
|  | 04-F | GGCTCCACGACTGGAAATGA |
|  | 04-R | CCAAGCTACGCAGTGCTGAT |
|  | 05-F | GAGAAAACGCTGCCCCAATG |
|  | 05-R | GATGATGTCTCCCTCGTGCC |
|  | 06-F | TGGCGTGACAAGTTCCTCTC |
|  | 06-R | GAAGTCGCTCTCCTCCTTCG |
|  | 07-F | TTAAGGGTTCGCGGGAAGAG |
|  | 07-R | GCCCTGTATAGTTGAGGGCAC |
|  | 08-F | TGGCGCGACAAATTCTTGTG |
|  | 08-R | AGTGGTTGAGTTCGTTGGCA |
| Promoter | SPX8pro-Sac1-F | CCGAGCTCAGTGTATGTAAAGATGC |
| analysis | SPX8pro-SaL1-R | TTGTCGACGGCTTTCCGTTAGCAAA |
| RNAi | SPX8-Xho1-Xba1-F | TCTCTAGACTCGAGGGTTCGCGGGAAGAGATGAT |
|  | SPX8-Sac1-BamH1-R | CAGAGCTCGGATCCTCTGAATGAAGGGCAAGCGA |
| Overexpression | SPX8-Pst1-F | CTCCTGCAGCATGAAATTCGGGAAGAGCCT |
|  | SPX8-EcoR1-R | GAGGAATTCTTACTTGGCTGCTTGTTCCA |
| Y2H verify | SPX8-BD-BamH1-F | GAGGATCCCGATGAAATTCGGGAAG |
|  | SPX8-BD-Pst1-R | CGGACGTCTTTACTTGGCTGCTTGT |
|  | PTF1-AD-Nde1-F | AACCATATGATGGACCAAGCTCCGGGAGG |
|  | PTF1-AD-Sma1-R | GATCCCGGGTGTCAGAGCTCCATCTTCAATT |
| BIFC | SPX8-Xba1-F | AGCTCTAGAATGAAATTCGGGAAGAGCCT |
|  | SPX8-Pac1-R | GGTTAATTAACCTTGGCTGCTTGTTCCAGAA |
|  | PTF1-xba1-F | AGCTCTAGAATGGACCAAGCTCCGGGAGG |
|  | PTF1-Pac1-R | GGGTTAATTAACGAGCTCCATCTTCAATTGAT |
| Pull down | SPX8-BamH1-F | AGCGGATCCATGAAATTCGGGAAGAGCCT |
|  | SPX8-Sal1-R | TTGGTCGACTGCTTGGCTGCTTGTTCCAGAA |
|  | PTF1-Sal1-F | AGAGTCGACATATGGACCAAGCTCCGGGAGG |
|  | PTF1-Sal1-R | GCGGTCGACTCGAGCTCCATCTTCAATTGAT |
| Transcriptional | SPX8-AD-Nde1-F | AACCATATGATGAAATTCGGGAAGAGCCTGA |
| activation | SPX8-AD-Sma1-R | TTCCCGGGTTTACTTGGCTGCTTGTTCCAGA |
|  | PTF1-BD-Nde1-F | AACCATATGATGGACCAAGCTCCGGGAGG |
|  | PTF1-BD-Sma1-R | GATCCCGGGTGTCAGAGCTCCATCTTCAATT |
